# Genetic diversity of medically important and emerging *Candida* species causing invasive infection

**DOI:** 10.1186/s12879-015-0793-3

**Published:** 2015-02-13

**Authors:** Karina Bellinghausen Merseguel, Angela Satie Nishikaku, Anderson Messias Rodrigues, Ana Carolina Padovan, Renata Carmona e Ferreira, Analy Salles de Azevedo Melo, Marcelo Ribeiro da Silva Briones, Arnaldo Lopes Colombo

**Affiliations:** Laboratório Especial de Micologia (LEMI), Disciplina de Infectologia, Departamento de Medicina, Universidade Federal de São Paulo, São Paulo, Rua Pedro de Toledo, 669, quinto andar, Edifício de Pesquisas II, Zipcode 04039-032 São Paulo, SP Brazil; Departamento de Microbiologia, Imunologia e Parasitologia, Disciplina de Biologia Celular, Universidade Federal de São Paulo, São Paulo, Brazil; Departamento de Microbiologia e Imunologia, Instituto de Ciências Biomédicas, Universidade Federal de Alfenas, Alfenas, MG Brazil; Laboratório de Genômica Evolutiva e Biocomplexidade, Departamento de Microbiologia, Imunologia e Parasitologia, Universidade Federal de São Paulo, São Paulo, Brazil

**Keywords:** *Candida* spp., DNA sequencing, Internal transcribed spacer region, Haplotype network, Nosocomial acquisition of candidemia

## Abstract

**Background:**

Genetic variation in the ribosomal DNA (rDNA) internal transcribed spacer (ITS) region has been studied among fungi. However, the numbers of ITS sequence polymorphisms in the various *Candida* species and their associations with sources of invasive fungal infections remain poorly investigated. Here, we characterized the intraspecific and interspecific ITS diversity of *Candida* spp. strains collected from patients with bloodstream or oroesophageal candidiasis.

**Methods:**

We selected cultures of representative medically important species of *Candida* as well as some rare and emerging pathogens. Identification was performed by micromorphology and by biochemical testing using an ID32C^®^ system, as well as by the sequencing of rDNA ITS. The presence of intraspecific ITS polymorphisms was characterized based on haplotype networks, and interspecific diversity was characterized based on Bayesian phylogenetic analysis.

**Results:**

Among 300 *Candida* strains, we identified 76 *C. albicans*, 14 *C. dubliniensis*, 40 *C. tropicalis*, 47 *C. glabrata*, 34 *C. parapsilosis* (*sensu stricto*), 31 *C. orthopsilosis*, 3 *C. metapsilosis,* 21 *Meyerozyma guilliermondii* (*C. guilliermondii*), 12 *Pichia kudriavzevii* (*C. krusei*), 6 *Clavispora lusitaniae* (*C. lusitaniae*), 3 *C. intermedia*, 6 *Wickerhamomyces anomalus* (*C. pelliculosa*), and 2 *C. haemulonii* strains, and 1 *C. duobushaemulonii*, 1 *Kluyveromyces marxianus* (*C. kefyr*), 1 *Meyerozyma caribbica* (*C. fermentati*), 1 *Pichia norvegensis* (*C. norvegensis*), and 1 *Lodderomyces elongisporus* strain*.* Out of a total of seven isolates with inconsistent ID32C^®^ profiles, ITS sequencing identified one *C. lusitaniae* strain, three *C. intermedia* strains, two *C. haemulonii* strains and one *C. duobushaemulonii* strain. Analysis of ITS variability revealed a greater number of haplotypes among *C. albicans*, *C. tropicalis*, *C. glabrata* and *C. lusitaniae*, which are predominantly related to endogenous sources of acquisition. Bayesian analysis confirmed the major phylogenetic relationships among the isolates and the molecular identification of the different *Candida* spp.

**Conclusions:**

Molecular studies based on ITS sequencing are necessary to identify closely related and emerging species. Polymorphism analysis of the ITS rDNA region demonstrated its utility as a genetic marker for species identification and phylogenetic relationships as well as for drawing inferences concerning the natural history of hematogenous infections caused by medically important and emerging *Candida* species.

**Electronic supplementary material:**

The online version of this article (doi:10.1186/s12879-015-0793-3) contains supplementary material, which is available to authorized users.

## Background

Invasive candidiasis is recognized as a cause of morbidity and mortality in tertiary care hospitals worldwide [1,2]. Although the majority of cases of invasive yeast infection are attributed to *Candida albicans*, there are increasing rates of infection by non-*C. albicans* species in various parts of the world [2,3]. Conventional methods used by reference centers for the identification of medically important yeasts have been progressively replaced by PCR-based methods and proteomics [4,5]. However, in resource-limited settings, commercial biochemical tests still represent the cornerstone for the identification of human yeast pathogens [4]. These methods are usually time-consuming and have potential limitations with respect to the accurate identification of rare pathogens and of cryptic species of *Candida* [6].

The establishment of DNA sequencing as a gold standard method for yeast identification by clinical laboratories has been hindered by several factors, including its cost, the lack of well-trained professionals [7], the limitations of the currently accepted DNA barcode system for fungi [8], and poor standardization of quality controls to ensure the accuracy of molecular methods [9]. Other important issues include the quality of reference nucleotide sequences derived from well-characterized yeast collections deposited in public genomic databases and the limited number of fungal species, especially those related to human infections, for which data are present in sequence databases [10,11]. Nevertheless, progress in microorganism genomics and its application to the taxonomy of fungal pathogens has enabled an extensive review of several genera and the recognition of cryptic species within formerly recognized taxons, for example, the *C. parapsilosis* species complex, the *C. guilliermondii* complex, the *C. haemulonii* complex, *C. rugosa* and others [12-15]. Consequently, there is a need to expand public nucleotide databases to include sequences of emerging fungal pathogens [10,11].

Despite the limitations cited above, the utilization of the ribosomal DNA (rDNA) internal transcribed spacer (ITS) region for sequence analysis appears to be the most reliable strategy for the accurate and rapid molecular identification of fungal pathogens that infect humans [16,17]. Furthermore, polymorphisms in the ITS region have been extensively addressed in phylogenetic, taxonomic and population dynamics studies and are particularly useful for the delineation of *Candida* species and strains [16,18].

Haplotype analysis through the detection of single nucleotide polymorphisms (SNPs) found in particular target DNA sequences has been shown to be useful in the estimation of the intraspecies genetic diversity of fungal species, and this technique has also been used in population genetics studies of fungi that cause human and animal infections, permitting their evaluation from phylogenetic, biogeographic and epidemiologic perspectives [19-21].

Several molecular investigations characterizing the frequency of outbreaks and nosocomial clusters in patients with candidemia have been performed [22-25]. The methods used in these investigations have included pulsed-field gel electrophoresis (PFGE), random amplification of polymorphic DNA (RAPD), analysis of restriction fragment length polymorphisms (RFLP), PCR fingerprinting and multilocus sequence typing (MLST) [26-28]. In this context, analysis of nosocomial clustering of candidemia may be useful for determining the true frequency of exogenously acquired *Candida* infections transmitted to patients by the hands of caregivers and by contamination associated with invasive medical procedures.

In the present study, we aimed to determine the potential use of the rDNA ITS region as a molecular marker for evaluating genetic diversity within and among clinically important and emerging *Candida* species from a large Brazilian yeast collection characterized by conventional and molecular methods. The presence of intraspecific ITS variability was characterized based on haplotype networks, and Bayesian analysis was used to develop phylogenetic inferences. The incorporation of ITS sequences of human pathogenic *Candida* species into public nucleotide sequence databases and the reliability of ITS sequence data were also addressed.

## Methods

### Selection of microorganisms

For this study, 300 strains of *Candida* spp. were selected from the large yeast stock culture collection of the Laboratório Especial de Micologia, Escola Paulista de Medicina, Universidade Federal de São Paulo, Brazil. All fungal isolates were collected between 1997 and 2011 during multicenter surveillance studies conducted at Brazilian medical centers [1,29-31]. We selected cultures of representative medically important species of *Candida*, as well as cultures of some rare or emerging pathogens. With the exception of *C. dubliniensis* strains (n = 14), which were isolated from patients with oroesophageal infection, all species (n = 286) were obtained from blood cultures of patients with fungemia. In addition, the following 11 reference/type strains were included: *C. albicans* SC5314, *C. albicans* ATCC 24433, *C. glabrata* ATCC 2001, *C. lusitaniae* ATCC 66035, *C. krusei* ATCC 6258, *C. tropicalis* ATCC 13803*, C. parapsilosis* ATCC 22019, *C. orthopsilosis* ATCC 96141, *C. metapsilosis* ATCC 96143, *C. guilliermondii* CBS 566 and *C. dubliniensis* CBS 7987. The clinical isolates and the reference/type strains were identified simultaneously using phenotypic and molecular methods. In addition to the nucleotide sequences of the reference/type strains obtained and described above, the sequences of the following eight strains were used for haplotype and phylogenetic analyses: *Lodderomyces elongisporus* ATCC 11503 (GenBank accession number: NR_111593.1), *C. kefyr* ATCC 60480 (GenBank accession number: GU256755.1), *C. pelliculosa* CBS 606 (MycoBank accession number: 346023), *C. fermentati* CBS 2022 (GenBank accession number: EU568913.1), *C. norvegensis* CBS 2128 (GenBank accession number: AB278167.1), *C. intermedia* WM 811 (GenBank accession number: EF568011.1), *C. haemulonii* CBS 10970 (GenBank accession number: JX459674.1) and *C. duobushaemulonii* CBS 7798 (GenBank accession number: JX459666.1). This work was approved by the institutional board on ethics in research of the Universidade Federal de São Paulo, Brazil (CEP008/11).

### Conventional identification of *Candida* species

Cultures of *Candida* spp. stored at -80°C were plated on CHROMagar^TM^*Candida* (CHROMagar Microbiology, Paris, France) prepared according to the manufacturer’s instructions for 48 h at 37°C to obtain pure colonies and for presumptive identification of *Candida* spp. Slide cultures on cornmeal agar medium with 1.2% Tween 80 were prepared to evaluate the presence of chlamydoconidia, blastoconidia and pseudohyphae. The ability to grow on Difco™ Sabouraud Dextrose Agar (SDA) (Becton Dickinson & Co. Sparks, MD, USA) plates at 42°C after 48 h of culture [32] or on hypertonic Sabouraud broth at 37°C for 96 h was used to discriminate between *C. albicans* and *C. dubliniensis* [32,33]. Reference strains of *C. albicans* (ATCC 24433) and *C. dubliniensis* (CBS 7987) were used as controls. With the exception of *C. albicans* and *C. dubliniensis*, the biochemical profiles of all *Candida* species were evaluated using a commercial ID32C^®^ system according to the manufacturer’s instructions (bioMérieux, Marcy-l’Étoile, France).

### Molecular identification of *Candida* species by sequencing of rDNA ITS

Total genomic DNA was extracted from the *Candida* isolates using PrepMan^®^ Ultra Sample Preparation Reagent (Applied Biosystems, Inc., Foster City, CA, USA) according to the manufacturer’s instructions. PCR for the amplification of the ITS region was performed using the forward primer V9G (5′-TTACGTCCCTGCCCTTTGTA-3′) and the reverse primer LS266 (5′-GCATTCCCAAACAACTCGACTC-3′) [34]. The total length of the amplified product was approximately 924 base pairs for *C. albicans*. A total reaction volume of 25 μl containing 40 ng/ml of genomic DNA, 10 pmol/μl of each primer, and PCR Master Mix with 50 units/ml of Taq DNA polymerase, 3 mM MgCl_2_, 400 μM dNTPs (Promega, Madison, WI, USA), and sterile water were used for the PCR reactions, which were performed in a Veriti 96-well Thermal Cycler (Applied Biosystems, Inc., Foster City, CA, USA) under the following conditions: an initial denaturation step at 94°C for 5 min, 35 cycles of denaturation at 94°C for 1 min, annealing at 56°C for 30 s, and extension at 72°C for 2 min, and a final extension step at 72°C for 10 min. Positive (DNA from *C. albicans* ATCC 24433) and negative (sample lacking DNA) controls were included in all assays. The amplicons were verified by electrophoresis at 90 volts in 1% agarose/SYBR^®^ Safe DNA Stain (Invitrogen, Carlsbad, CA, USA) gels and photographed using a UV transilluminator.

PCR products were subjected to dideoxynucleotide sequencing with a Big Dye Terminator Reaction Kit v3.1 (Applied Biosystems, Inc., Foster City, CA, USA), using the forward primers V9G and ITS1 (5′-TCCGTAGGTGAACCTGCGG-3′) and the reverse primers LS266 and ITS4 (5′-TCCTCCGCTTATTGATATGC-3′) [34,35] according to the manufacturer’s instructions. After purification and denaturation, the samples were run on an automated ABI 3130 genetic analyzer (Applied Biosystems, Inc., Foster City, CA, USA). For the sequencing reactions, a total of six sequences were used, including three forward strands (one strand sequenced with V9G and two with ITS1) and three reverse strands (one strand sequenced with LS266 and two with ITS4) for each strain to increase confidence in the sequencing data for the detection of nucleotide polymorphisms and to avoid experimental artifacts.

Consensus sequence assembly and editing were performed using the programs Phred/Phrap and the sequence editor Consed [36-38]. The error probability for each called base was assessed, considering a Phred score > 40, which was associated with a base call accuracy of 99.99%. High-quality consensus sequences were obtained for analysis with assembly errors of less than one per 100 base pairs after editing. The consensus sequences obtained in our study were aligned and compared with sequences deposited in public genomic databases (GenBank, NCBI, USA and CBS database, the Netherlands). To ensure the high accuracy of the results obtained using the nucleotide sequence alignment tools, an e-value of less than 10^-5^ and a maximum identity of equal to or higher than 98% were considered for the correct identification of *Candida* at the species level.

### Analysis of intraspecific and interspecific diversities of ITS sequences of *Candida* spp.

Haplotype analysis was conducted to assess the ITS intraspecific variability of the *Candida* species. In the present study, a haplotype was defined as a unique combination of SNPs along a sequence, i.e., each different sequence in an alignment. The ITS sequences of the *Candida* spp. were aligned and edited using the muscle algorithm implemented in SEAVIEW program 4.2.12 [39], excluding 18S and 28S rDNA. The complete ITS sequences, including ITS1 and ITS2 and the 5.8S region of rDNA were subjected to sequence polymorphism analysis using DnaSP version 5.10 software [40]. The analysis was based on the number of haplotypes (Hap), variable sites, haplotype diversity (Hd), and nucleotide diversity (Pi) [41]. In brief, haplotype diversity is a measure of the occurrence of a single haplotype in a given species, considering the number of sequences analyzed and the total number of haplotypes found. Values range from 0 to 1, with those closer to 1 indicating higher variability. Nucleotide diversity is a measure of the average number of nucleotide differences per site between two sequences. Haplotype 1 corresponded with the reference or type strain of each *Candida* spp.

For the ITS haplotype network, a total of 319 DNA sequences of clinical (n = 300) and reference/type strains (n = 19) comprising 17 *Candida* species and 1 non-*Candida* species (*Lodderomyces elongisporus*) were aligned. The haplotype network file (Roehl data file) was created using DnaSP v.5.10 software, considering gaps. The network was generated by the median-joining method [42] using Network v4.612 software (http://www.fluxus-engineering.com/).

To analyze the conservation of phylogenetic relationships among the different *Candida* species, sequences representative of each haplotype (Additional file 1: Table S1) and the reference sequence found in the comparisons with the public genomic databases were aligned and edited using the muscle algorithm implemented in SEAVIEW program 4.2.12 [39] and considering only the ITS1-ITS2 and 5.8S-rDNA regions. Phylogenetic inference was performed using MrBayes 3.02 [43] with the default priors as input and was run twice with four chains. The number of generations was 2 million, and data were saved every 100 generations along with the GTR (general time reversible) model, the shape of the gamma distribution parameters, and the proportions of invariant sites estimated during the run. Bootstrap analysis was conducted by evaluating 1,000 pseudoreplicates of the alignment with SEAVIEW program 4.2.12 [39] using the neighbor-joining method.

### Nucleotide sequence accession numbers

The rDNA ITS sequences of the clinical strains of *Candida* spp. and *Lodderomyces elongisporus* were deposited in the GenBank database with the accession numbers KC408939 to KC408999. For complete information including strain name, species name and accession numbers, see Additional file 1: Table S1.

## Results

In the present study, one hundred percent concordance between the phenotypic and molecular methods was obtained for a collection of clinically important yeast isolates containing representative numbers of strains (≥ 40 strains) as follows: *C. albicans* (n = 76), *C. tropicalis* (n = 40) and *C. glabrata* (n = 47) (Additional file 2: Table S2). Growth tests in hypertonic broth and at 42°C allowed for the presumptive identification of 14 *C. dubliniensis* and 76 *C. albicans* isolates, and ITS sequencing confirmed the identification of these two species, indicating 100% concordance (Additional file 2: Table S2). As expected, among 69 isolates phenotypically identified as *C. parapsilosis* (*sensu lato*), ITS sequencing distinguished 34 *C. parapsilosis* (*sensu stricto*), 31 *C. orthopsilosis* and three *C. metapsilosis* isolates and one *Lodderomyces elongisporus* isolate. A total of 22 isolates were identified as *C. guilliermondii* by biochemical tests, while DNA sequence analysis detected 21 *Meyerozyma guilliermondii* (teleomorph of *C. guilliermondii*) isolates and one *Meyerozyma caribbica* (teleomorph of *C. fermentati*) isolate.

Seven isolates that exhibited inconclusive results based on the ID32C^®^ system were identified by ITS sequencing as *C. lusitaniae* (n = 1), *C. intermedia* (n = 3), *C. haemulonii* (n = 2) and *C. duobushaemulonii* (n = 1). Of six isolates identified as *C. lusitaniae* by molecular methods, one showed an inconsistent profile with the biochemical test. ITS sequencing distinguished species within the *C. haemulonii* complex, including two *C. haemulonii* isolates and one *C. duobushaemulonii* isolate (see Additional file 2: Table S2)*.*

**Figure 1:**
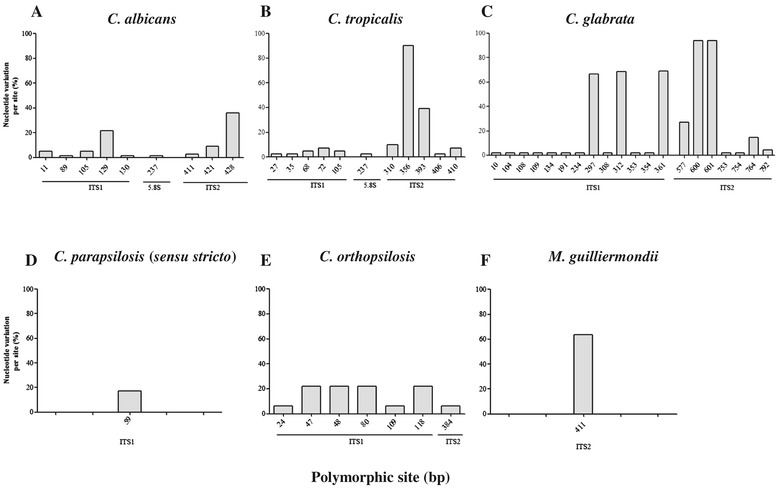
**Analysis of nucleotide sequence variation of rDNA ITS in medically important** ***Candida*** **spp.** Polymorphic sites in ITS1-5.8S-ITS2 rDNA were evaluated along the total sequence lengths for the following species for the assessment of the percentage of nucleotide variation per site, for which the sequences of the clinical strains were compared with the sequence of the reference or type strain for each species: **(A)** *C. albicans* (447 bp, 78 sequences), **(B)** *C. tropicalis* (437 bp, 41 sequences), **(C)** *C. glabrata* (803 bp, 48 sequences), **(D)** *C. parapsilosis* (*sensu stricto*) (430 bp, 35 sequences), **(E)** *C. orthopsilosis* (426 bp, 32 sequences), and **(F)** *M. guilliermondii* (516 bp, 22 sequences).

The presence of sequence polymorphisms along the total fragment length of the ITS rDNA (ITS1 and ITS2, including the 5.8S region) was analyzed in clinically important *Candida* species with representative numbers of strains (> 20 strains), as illustrated in Figure 1. A comparison of the ITS sequences of the clinical strains to the sequence of the reference or type strain of each *Candida* species revealed that nucleotide sequence variability per site was increased in ITS1 compared with ITS2. Five polymorphic sites in ITS1 and three in ITS2 were identified for *C. albicans* in addition to five variations in ITS1 and five in ITS2 for *C. tropicalis*, 13 in ITS1 and seven in ITS2 for *C. glabrata*, one in ITS1 for *C. parapsilosis* (*sensu stricto*), six in ITS1 and one in ITS2 for *C. orthopsilosis*, and one in ITS2 for *M. guilliermondii*. Sequence variations in the 5.8S region were detected only for *C. albicans* (one site) and *C. tropicalis* (one site).

Based on the results of rDNA ITS sequence analysis, we also determined the presence of intraspecific variability in the *Candida* species, of which three or more strains were analyzed and compared to type/reference strains (Table 1). High intraspecific variation was found for *C. albicans*, *C. tropicalis*, *C. glabrata*, *C. metapsilosis*, *P. kudriavzevii*, *C. lusitaniae* and *C. intermedia,* as evidenced by the large numbers of haplotypes and variable sites and haplotype (Hd = 0.6179 to 0.8571) and nucleotide (Pi = 0.00164 to 0.02815) diversity. It is worth mentioning that *C. lusitaniae* showed the highest haplotype diversity (Hd = 0.8571) despite the low number of isolates of this species (n = 6). The measurement of nucleotide diversity also revealed more nucleotide differences per site in *C. intermedia* (Pi = 0.02815) and *C. lusitaniae* (Pi = 0.02605) than in the other species tested.

**Table 1:** **Analysis of intraspecific variability of rDNA ITS sequences for clinically important and emerging** ***Candida*** **species**

***Candida*** **species**	**Number of sequences**	**Sequence length (base pair)**	**Number of haplotypes**	Haplotype diversity*	**Number of variable sites (ITS1-5.8S-ITS2)**	Nucleotide diversity** (Pi)
*Candida albicans*	78	447	12	0.7802	9	0.00190
*Candida dubliniensis*	15	454	2	0.1333	6	0.00088
*Candida glabrata*	48	803	11	0.6179	20	0.00290
*Candida tropicalis*	41	437	9	0.7500	11	0.00182
*Candida parapsilosis* (*sensu stricto*)	35	430	2	0.2924	1	0.00068
*Candida orthopsilosis*	32	426	3	0.4456	7	0.00083
*Candida metapsilosis*	3	443	3	0.8333	8	0.00830
Meyerozyma guilliermondii***	22	516	2	0.4848	1	0.00094
Pichia kudriavzevii***	13	421	5	0.7821	4	0.00164
Clavispora lusitaniae***	7	299	5	0.8571	17	0.02605
*Candida intermedia*	4	302	3	0.8333	18	0.02815
Wickerhamomyces anomalus***	7	523	1	0	0	0
*Candida haemulonii*	3	286	1	0	0	0

*The haplotype diversity is a measure of the presence of a single haplotype in a given species, considering the number of sequences analyzed and the total number of haplotypes found. This parameter varies from 0 to 1, with values of closer to 1 indicating higher variability.

**The nucleotide diversity (Pi) is a measure of the average number of nucleotide differences per site between two sequences.

***Meyerozyma guilliermondii = teleomorph of *Candida guilliermondii*, *Pichia kudriavzevii* = teleomorph of *Candida krusei*, *Clavispora lusitaniae* = teleomorph of *Candida lusitaniae*, and *Wickerhamomyces anomalus =* teleomorph of *Candida pelliculosa.*

On the other hand, minor intraspecific variations were observed among isolates of *C. parapsilosis* (*sensu stricto*) (Hd = 0.2924 and Pi = 0.00068), *C. orthopsilosis* (Hd = 0.4456 and Pi = 0.00083), and *M. guilliermondii* (Hd = 0.4848 and Pi = 0.00094). Another interesting finding was the presence of only one ITS haplotype for each of *Wickerhamomyces anomalus* (n = 7) and *C. haemulonii* (n = 3), as demonstrated by the lack of polymorphic sites and haplotype and nucleotide diversities in these species.

**Figure 2:**
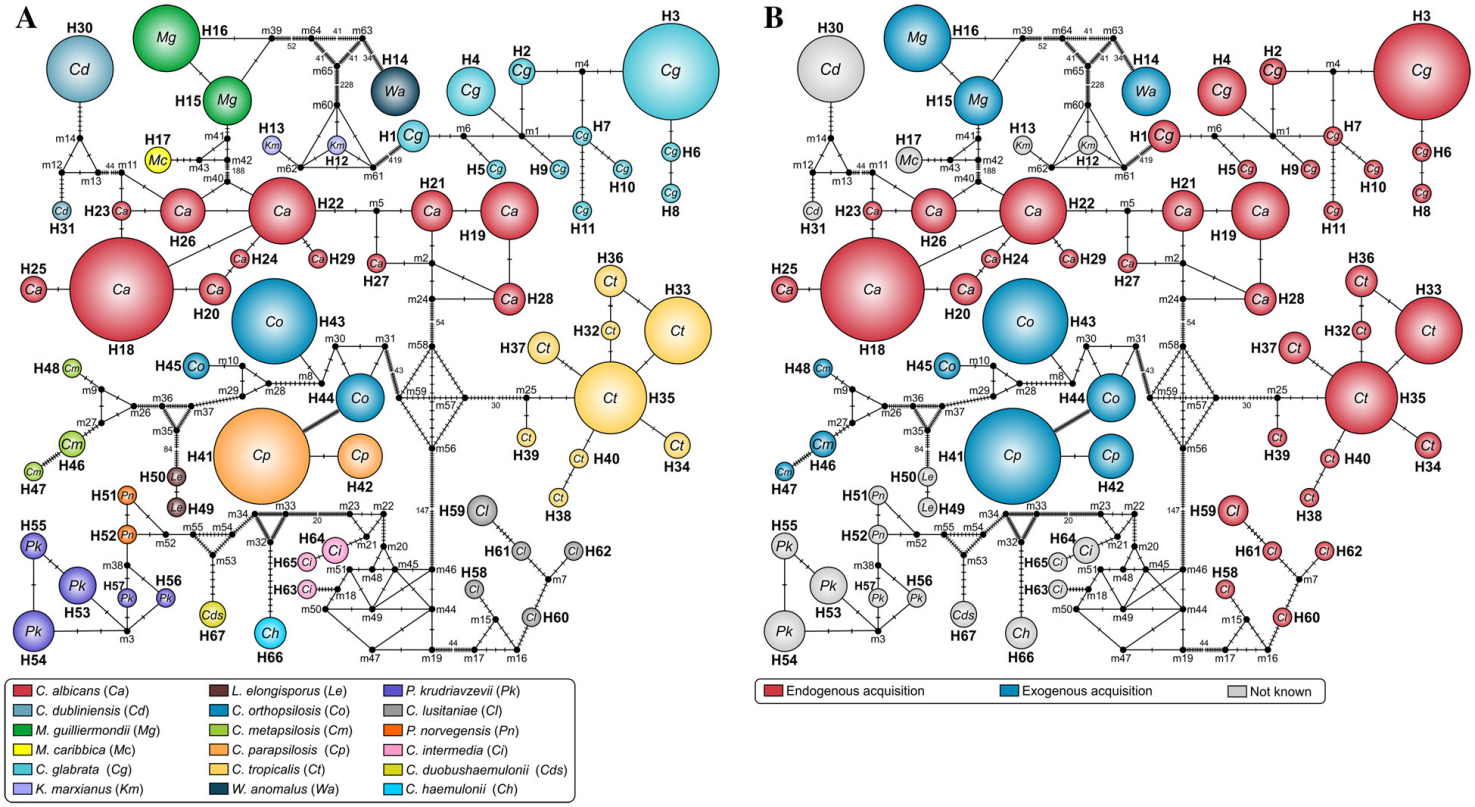
**Genetic diversity of medically important and emerging** ***Candida*** **spp. based on rDNA ITS sequences. (A)** The median-joining haplotype network of 319 DNA sequences of clinical (n = 300) and reference/type strains (n = 19) belonging to 17 *Candida* species and 1 non-*Candida* species (*Lodderomyces elongisporus*). Each circle represents one haplotype (H1-H67), and the circle circumference is proportional to the haplotype frequency of the dataset. The black dots (median vectors, m) represent unsampled or extinct haplotypes in the population. Mutational steps are represented by lines between haplotypes and, in cases of long branches, by values. **(B)** The haplotypes are color-coded, and each color represents the predominantly associated source of acquired infection for the haplotype (endogenous *versus* exogenous).

The ITS haplotype network constructed by the alignment of 319 sequences (317 sequences of *Candida* spp. and 2 *L. elongisporus* sequences) showed the presence of 67 haplotypes (Figure 2A). At a total of 873 sites analyzed, we found 716 variable positions (Hd = 0.9556), considering sites with gaps. Notable genetic diversity was observed, as demonstrated by the large numbers of haplotypes in clinically relevant species and in some rare species of *Candida*, including 12 *C. albicans* (n = 78), 9 *C. tropicalis* (n = 41), 11 *C. glabrata* (n = 48), 5 *P. kudriavzevii* (n = 13), 5 *C. lusitaniae* (n = 7) and 3 *C. intermedia* haplotypes (n = 4). In contrast, only 2 haplotypes each were found for *C. parapsilosis* (*sensu stricto*) (n = 35*)*, *C. dubliniensis* (n = 15) and *M. guilliermondii* (n = 22), and only 1 haplotype each was found for *W. anomalus* (n = 7) and *C. haemulonii* (n = 3). With respect to acquisition sources of *Candida* infection (Figure 2B), high ITS diversity was found among species predominantly associated with endogenous sources of infection (*C. albicans*, *C. tropicalis*, *C. glabrata* and *C. lusitaniae*), while low genetic diversity was observed in species predominantly related to exogenous infection sources (*C. parapsilosis* species complex, *M. guilliermondii* and *W. anomalus*).

**Figure 3:**
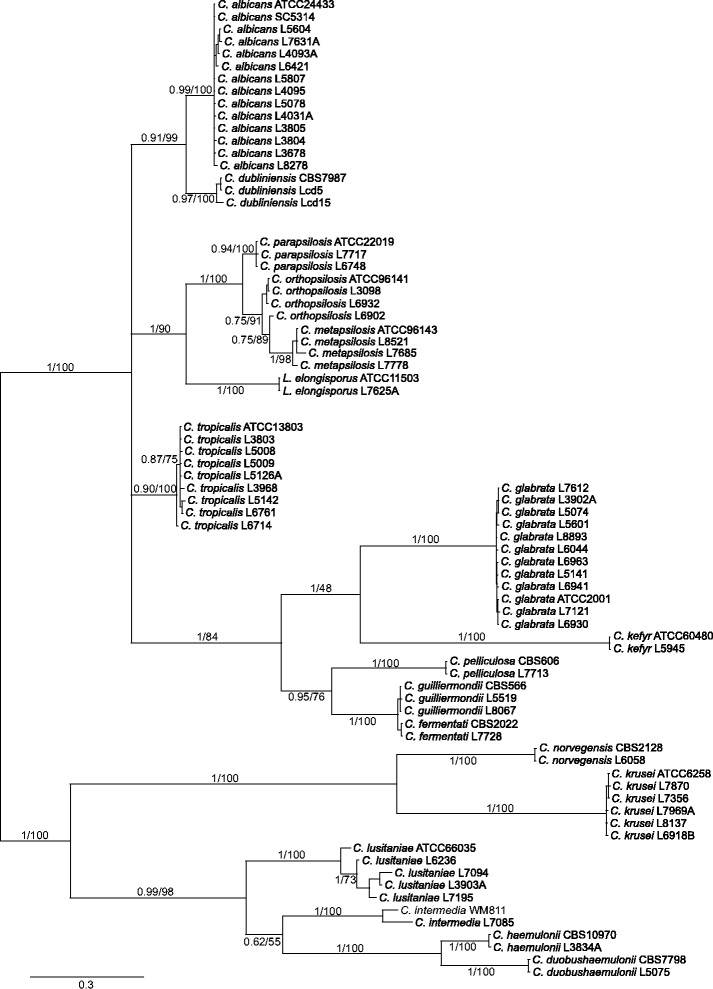
**Unrooted Bayesian consensus phylogenetic tree of rDNA ITS haplotype representatives of** ***Candida*** **spp.** Species names were identified according to anamorphic nomenclature. Posterior probabilities and bootstrap values are depicted in the main nodes (pp/bootstrap). The Bayesian tree was inferred from 2 million generations with a 50% burn-in, and runs were saved every 100 generations. The average standard deviation of the split frequencies was 0.008203. The selected model was fA = 0.274572, fC = 0.203973, fG = 0.223583, and fT = 0.297972 with the rate matrix [A–C] = 0.151397, [A–G] = 0.142740, [A–T] = 0.199583, [C–G] = 0.112413, [C–T] = 0.269359 and [G–T] = 0.124502. The shape parameter of the gamma distribution was alpha = 1.517708, and the proportion of invariant sites was I = 0.196504.

Bayesian analysis (Figure 3) confirmed the molecular identification of and the major phylogenetic relationships among the various *Candida* spp. isolates, with high posterior probabilities and bootstrap values in the branches. Intraspecies variation was also observed within the clades, demonstrating the conservation of the haplotypes described herein.

In our study, large numbers of ITS sequences were generated based on quality control criteria designed to ensure the accuracy of the molecular data used to identify the *Candida* isolates at the species level. The quality control criteria included PCR controls, the use of triplicate reactions with both DNA strands for sequencing, the estimation of assembly errors for high-quality consensus sequences, and BLAST search parameters. We deposited 60 ITS sequences of the most important fungal pathogens and rare or emerging pathogens, including 17 *Candida* species and 1 non-*Candida* species (*Lodderomyces elongisporus*), in the NCBI database (GenBank, USA). Fifty-eight ITS sequences, comprising the sequences of all *Candida* species and *L. elongisporus* submitted to GenBank, were deposited in the ISHAM-ITS reference database, which can be accessed at http://www.isham.org or at http://its.mycologylab.org.

## Discussion

The accurate identification of *Candida* spp. is essential for selecting the most effective therapeutic strategies to control invasive fungal infections caused by these species [7]. Here, the phenotypic methods used performed well in identifying clinically important *Candida* species, including *C. albicans*, *C. tropicalis*, the *C. parapsilosis* complex and *C. glabrata*. However, the ID32C^®^ system failed to identify one *C. lusitaniae* isolate, three *C. intermedia* isolates and three isolates belonging to the *C. haemulonii* species complex. Some limitations of conventional methods have been described for discrimination between *C. lusitaniae* and the closely related species *C. pulcherrima* [44]. Conventional identification of the *C. haemulonii* species complex is still limited, however, because it is not included in the database of current commercial biochemical systems, such as ID32C^®^ [6]. Nevertheless, the reliable identification of rare and emerging *Candida* species that cause hematogenous infection, such as *C. lusitaniae*, *C. intermedia* and the *C. haemulonii* complex, was possible using DNA sequencing, corroborating with the results presented by other authors [6,44,45].

For epidemiological or diagnostic purposes, DNA-based methods have been used to differentiate the species forming the *C. parapsilosis* complex [46]. Out of a total of 69 isolates phenotypically identified as *C. parapsilosis* (*sensu lato*), 34 were determined to be *C. parapsilosis* (*sensu stricto*), 31 were found to be *C. orthopsilosis*, three were found to be *C. metapsilosis* and one was determined to be *Lodderomyces elongisporus* by ITS sequencing. *Lodderomyces elongisporus* was initially described as a teleomorph of *C. parapsilosis* [47]. Although *L. elongisporus* is considered to be an uncommon cause of human disease, isolates have been identified by DNA sequence analysis of some clinical sources [6,48]. *M. guilliermondii* is also currently considered to comprise a complex formed by *Debaryomyces hansenii* (teleomorph of *C. famata*), *M. caribbica*, *C. carpophila* and *C. xestobii* [13], which can be differentiated by molecular methods, including sequencing of the ITS and D1/D2 rDNA regions [49]. Here, we found one isolate of *M. caribbica* by sequence analysis, whereas only *M. guilliermondii* isolates were detected by biochemical testing.

We also investigated intraspecific rDNA ITS polymorphisms of isolates from 17 *Candida* species and 1 non-*Candida* species (*Lodderomyces elongisporus*). Some authors have addressed the degree of variability within the rDNA ITS region among fungi and have also observed higher intraspecific diversity within the ITS1 region than within the ITS2 region [50,51]. Based on haplotype and network analysis, a high intraspecific variability of ITS sequences was observed in species that primarily represent endogenous sources of infection, including *C. albicans*, *C. tropicalis*, *C. glabrata* and *C. lusitaniae* [2]. Species with high genetic diversity are most frequently human commensals, and this finding could explain the existence of additional genetic adaptation within normal microbiota with older evolutionary origins. Although the natural mode of reproduction of *C. albicans* is known to be clonal, other mechanisms that increase the genetic variability of this species could occur, including recombination [52,53]. Pfaller et al. [54] have tested 47 *C. lusitaniae* isolates, obtaining 28 different karyotype profiles and 25 different types of restriction endonuclease analysis of genomic DNA (REAG) profiles. Our data confirm the great diversity among *C. lusitaniae* haplotypes, possibly indicating the existence of a non-clonal form of propagation. In contrast, less intraspecific variation was observed in the isolates of *C. parapsilosis* and *C. orthopsilosis*, in which the primary mode of infection is thought to be exogenous [55]. Previous studies have shown a lower sequence variability of *C. parapsilosis* (*sensu stricto*) compared with *C. orthopsilosis* and *C. metapsilosis* isolates [56,57]. Furthermore, we detected no ITS polymorphisms in six *W. anomalus* isolates. Barchiesi and colleagues [58] have studied 46 clinical isolates of *W. anomalus* using RFLP and RAPD and have found that all of these isolates produce similar band patterns. The lack of ITS variability of *W. anomalus* strains could be explained by the small number of sequenced isolates (n = 6), by possible clonal origin or even by a low mutation rate of the gene selected in our present study. In contrast with species that are more prevalent as human commensal organisms, other *Candida* species, such as the *C. parapsilosis* complex, *M. guilliermondii* and *W. anomalus,* have been reported to be exogenous organisms that have been isolated from environmental sources (plants, soil, insects, and food), medical devices (central venous catheter and parenteral nutrition), or the hands of health care workers [2,55,59,60]. Although they are considered to be rare causative agents of fungemia, *W. anomalus* and *M. guilliermondii* have been associated with the occurrence of nosocomial outbreaks and pseudo-outbreaks, respectively, especially in pediatric intensive care units [61,62]. Species with low genetic variability that are more often associated with exogenous transmission could be less well adapted to human hosts, may predominantly undergo clonal reproduction, and might have recently diverged during their evolutionary histories [12,58,63].

In the present study, despite the limitation conferred by the use of only one genetic marker to estimate intraspecific diversity, it was possible to use the ITS haplotype network to identify remarkable genetic polymorphisms among clinical isolates comprising 17 *Candida* species. Of course, the accuracy of haplotype analysis is influenced by the molecular markers selected as well as by the number of strains in the database and the network methods used [64]. Importantly, all of the ITS sequences used for the evaluation of intraspecific genetic variation were generated by the direct sequencing of PCR products. The provision of reliable ITS sequences was based on quality control criteria that were applied from PCR to sequence analysis, for example, the amplification of products with a total length that included the entire ITS region, the use of a cut-off number of sequences to generate the consensus sequence for each strain, and the use of well-established computational tools for sequence assembly and editing.

Recent molecular epidemiological surveys aiming to assess genetic relationships among strains and temporal and geographic distributions of clustered isolates as well as to identify outbreaks and the recurrence of BSIs investigated the occurrence of the nosocomial clustering of candidemia caused by the most prevalent species of *Candida* [23,25]. Using PCR fingerprinting, a population-based study conducted in Iceland has shown that 18.7% to 39.9% of all cases of candidemia are nosocomial clusters primarily caused by *C. albicans*, *C. tropicalis* and *C. parapsilosis* [23]. In a recent epidemiologic study conducted by Maganti and colleagues [25], nosocomial clusters have been shown to represent 33% of total isolates causing candidemia in Canadian hospitals. Using MLST analysis, the genetic relatedness of *Candida* isolates has been assessed in two Brazilian studies of candidemia, in which different clusters have been found among isolates of *C. albicans* [24] and *C. tropicalis* [65]. Our present study reinforces the utility of sequence polymorphism analysis for evaluating the relationships among *Candida* species and its application for examining the molecular epidemiology of fungal diseases.

Comparative analysis of our *Candida* ITS sequences with those deposited in public genomic databases allowed for the definition of appropriate quality parameters for use with the sequence alignment search tools of the NCBI and CBS databases, as demonstrated by the high identities of our sequences at the species level to the sequences in the nucleotide repositories. However, some caution must be exercised with respect to species identification using DNA sequencing because several factors may influence the accuracy of this molecular assay, including the methodologies and programs used for sequence analysis and the limited number of representative pathogenic fungal species deposited in genomic databases, which may include incomplete sequences as well as errors in species nomenclature. Analysis of the ITS sequence regions of different taxonomic groups revealed that up to 20% of sequences deposited in GenBank may contain errors in species identification and/or outdated nomenclature and may lack descriptive and updated annotations [10].

## Conclusions

In conclusion, although conventional methods can be used to reliably identify the most common *Candida* species, molecular studies based on ITS sequencing are necessary for the identification of closely related and emerging species. DNA sequence polymorphisms, especially those observed in ITS1, were more likely to be found in *Candida* species primarily involved in endogenously acquired infections. Thus, *C. albicans, C. tropicalis, C. glabrata* and *C. lusitaniae* showed high genetic diversity, while species predominantly associated with exogenously acquired infections, such as *C. parapsilosis*, *M. guilliermondii* and *W. anomalus*, showed low intraspecific variability. In addition, our findings indicate the importance of generating accurate ITS sequence regions based on quantitative parameters, such as those used in this study. The use of this criterion may help to increase the number of good-quality DNA sequences of *Candida* species deposited in public genomic databases, ensuring reliable information for future studies involving the epidemiological, clinical and molecular characterization of opportunistic fungi.

## Additional files

Additional file 1: Table S1. Ribosomal DNA ITS sequences of *Candida* spp. and *Lodderomyces elongisporus* deposited in public sequence databases. Species names, strains, haplotypes and GenBank accession numbers. Additional file 2: Table S2. Comparative analysis of the phenotypic and molecular methods used to identify *Candida* species. The yeast strains were isolated from bloodstream (n = 286) and oroesophageal (n = 14) infections.

## Abbreviations

ATCC: American Type Culture Collection
BLASTn: Nucleotide Basic Local Alignment Search Tool
CBS: Centraalbureau voor Schimmelcultures
Hap: Haplotype
Hd: Haplotype diversity
ITS: Internal transcribed spacer
NCBI: National Center for Biotechnology Information
PCR: Polymerase chain reaction
rDNA: ribosomal DNA
rRNA: Ribosomal RNA
RAPD: Random amplification of polymorphic DNA
RFLP: Restriction fragment length polymorphisms
MLST: Multilocus sequence typing
PFGE: Pulsed-field gel electrophoresis
REAG: Restriction endonuclease analysis of genomic DNA
BSI: Bloodstream infection
MALDI-TOF MS: Matrix-assisted laser desorption/ionization-time of flight
GTR: General time reversible
