# Supplementary material for: Genetic diversity of medically important and emerging Candida species causing invasive infection
Source: BMC Infect Dis. 2015 Feb 13;15:57. doi: 10.1186/s12879-015-0793-3 (PMC4339437; doi:10.1186/s12879-015-0793-3)
Supplement: Additional file 1: Table S1. — Ribosomal DNA ITS sequences of Candida spp. and Lodderomyces elongisporus deposited in public sequence databases. Species names, strains, haplotypes and GenBank accession numbers. [file 12879_2015_793_MOESM1_ESM.doc]

**Additional File 1**

**Table S1 Ribosomal DNA ITS sequences of *Candida* spp. and *Lodderomyces elongisporus* deposited in public sequence database.** Species names, strains, haplotypes and GenBank accession numbers.

| ***Candida species*** | **Strain numbers** | **GenBank**  **accession numbers** | **Haplotype number** |
| --- | --- | --- | --- |
| *Candida dubliniensis* | Lcd5 | KC408939 | Cdubl_haplotype1 |
| *Candida dubliniensis* | Lcd15 | KC408940 | Cdubl_haplotype2 |
| *Candida albicans* | L5807 | KC408941 | Calb_haplotype1 |
| *Candida albicans* | L5604 | KC408942 | Calb_haplotype2 |
| *Candida albicans* | L4095 | KC408943 | Calb_haplotype3 |
| *Candida albicans* | L4093A | KC408944 | Calb_haplotype4 |
| *Candida albicans* | L5078 | KC408945 | Calb_haplotype5 |
| *Candida albicans* | L4031A | KC408946 | Calb_haplotype6 |
| *Candida albicans* | L3805 | KC408948 | Calb_haplotype7 |
| *Candida albicans* | L3804 | KC408949 | Calb_haplotype8 |
| *Candida albicans* | L3678 | KC408950 | Calb_haplotype9 |
| *Candida albicans* | L6421 | KC408951 | Calb_haplotype10 |
| *Candida albicans* | L7631A | KC408952 | Calb_haplotype11 |
| *Candida albicans* | L8278 | KC408953 | Calb_haplotype12 |
| *Candida glabrata* | L6930 | KC408954 | Cglab_haplotype1 |
| *Candida glabrata* | L3902A | KC408955 | Cglab_haplotype2 |
| *Candida glabrata* | L5074 | KC408956 | Cglab_haplotype3 |
| *Candida glabrata* | L5141 | KC408957 | Cglab_haplotype4 |
| *Candida glabrata* | L6044 | KC408958 | Cglab_haplotype5 |
| *Candida glabrata* | L6941 | KC408959 | Cglab_haplotype6 |
| *Candida glabrata* | L6963 | KC408960 | Cglab_haplotype7 |
| *Candida glabrata* | L7121 | KC408961 | Cglab_haplotype8 |
| *Candida glabrata* | L7612 | KC408962 | Cglab_haplotype9 |
| *Candida glabrata* | L8893 | KC408963 | Cglab_haplotype10 |
| *Candida glabrata* | L5601 | KC408964 | Cglab_haplotype11 |
| *Candida tropicalis* | L3803 | KC408965 | Ctrop_haplotype2 |
| *Candida tropicalis* | L5008 | KC408966 | Ctrop_haplotype3 |
| *Candida tropicalis* | L5009 | KC408967 | Ctrop_haplotype4 |
| *Candida tropicalis* | L5126A | KC408968 | Ctrop_haplotype5 |
| *Candida tropicalis* | L3968 | KC408969 | Ctrop_haplotype6 |
| *Candida tropicalis* | L5142 | KC408970 | Ctrop_haplotype7 |
| *Candida tropicalis* | L6714 | KC408971 | Ctrop_haplotype8 |
| *Candida tropicalis* | L6761 | KC408972 | Ctrop_haplotype9 |
| *Meyerozyma guilliermondii* | L8067 | KC408973 | *Candida guilliermondii**; Cguill_haplotype1 |
| *Meyerozyma guilliermondii* | L5519 | KC408974 | *Candida guilliermondii**; Cguill_haplotype2 |
| *Pichia kudriavzevii* | L7356 | KC408975 | *Candida krusei**; Ckr_haplotype1 |
| ***Candida species*** | **Strain numbers** | **GenBank**  **accession numbers** | **Haplotype number** |
| *Pichia kudriavzevii* | L7969A | KC408976 | *Candida krusei**; Ckr_haplotype2 |
| *Pichia kudriavzevii* | L7870 | KC408977 | *Candida krusei**; Ckr_haplotype3 |
| *Pichia kudriavzevii* | L8137 | KC408978 | *Candida krusei**; Ckr_haplotype4 |
| *Pichia kudriavzevii* | L6918B | KC408979 | *Candida krusei**; Ckr_haplotype5 |
| *Clavispora lusitaniae* | L3903A | KC408980 | *Candida lusitaniae**; Clu_haplotype2 |
| *Clavispora lusitaniae* | L7094 | KC408981 | *Candida lusitaniae**; Clu_haplotype3 |
| *Clavispora lusitaniae* | L6236 | KC408982 | *Candida lusitaniae**; Clu_haplotype4 |
| *Clavispora lusitaniae* | L7195 | KC408983 | *Candida lusitaniae**; Clu_haplotype5 |
| *Wickerhamomyces anomalus* | L7713 | KC408984 | *Candida pelliculosa**; Cpell_haplotype1 |
| *Candida parapsilosis* (*sensu stricto*) | L6748 | KC408985 | Cpara_haplotype1 |
| *Candida parapsilosis* (*sensu stricto*) | L7717 | KC408986 | Cpara_haplotype2 |
| *Candida orthopsilosis* | L3098 | KC408987 | Corth_haplotype1 |
| *Candida orthopsilosis* | L6932 | KC408988 | Corth_haplotype2 |
| *Candida orthopsilosis* | L6902 | KC408989 | Corth_haplotype3 |
| *Candida metapsilosis* | L8521 | KC408990 | Cmet_haplotype1 |
| *Candida metapsilosis* | L7685 | KC408991 | Cmet_haplotype2 |
| *Candida metapsilosis* | L7778 | KC408992 | Cmet_haplotype3 |
| *Candida haemulonii* | L3834A | KC408993 | *C. haemulonii* complex; Chaem_haplotype1 |
| *Candida duobushaemulonii* | L5075 | KC408994 | *C. haemulonii* complex; Cdhaem_haplotype1 |
| *Candida intermedia* | L7085 | KC408995 | Cinter_haplotype2 |
| *Meyerozyma caribbica* | L7728 | KC408996 | *Candida fermentati**; Pcarib_haplotype1 |
| *Kluyveromyces marxianus* | L5945 | KC408997 | *Candida kefyr**; Ckef_haplotype2 |
| *Pichia norvegensis* | L6058 | KC408998 | *Candida norvegensis**; Cnor_haplotype2 |
| *Lodderomyces elongisporus* | L7625A | KC408999 | Lelong_haplotype1 |

We also included the anamorphic nomenclature (*) and the haplotype numbers of *Candida* species and *L. elongisporus* referred in the GenBank.
