# Supplementary material for: Genetic diversity of medically important and emerging Candida species causing invasive infection
Source: BMC Infect Dis. 2015 Feb 13;15:57. doi: 10.1186/s12879-015-0793-3 (PMC4339437; doi:10.1186/s12879-015-0793-3)
Supplement: Additional file 2: Table S2. — Comparative analysis of the phenotypic and molecular methods used to identify Candida species. The yeast strains were isolated from bloodstream (n = 286) and oroesophageal (n = 14) infections. [file 12879_2015_793_MOESM2_ESM.doc]

**Additional File 2**

**Table S2 Comparative analysis of the phenotypic and molecular methods used to identify *Candida* species. The yeast strains were isolated from bloodstream (n = 286) and oroesophageal (n = 14) infections.**

| **Phenotypic identification of *Candida* species** | **Number of isolates** | **Molecular identification of *Candida* species** | | **Number of isolates** | |  |
| --- | --- | --- | --- | --- | --- | --- |
| *Candida albicans** | 76 | | *Candida albicans* | | 76 | |
| *Candida dubliniensis** | 14 | | *Candida dubliniensis* | | 14 | |
| *Candida glabrata* | 47 | | *Candida glabrata* | | 47 | |
| *Candida tropicalis* | 40 | | *Candida tropicalis* | | 40 | |
| *Candida krusei* | 12 | | *Pichia kudriavzevii*** | | 12 | |
| *Candida lusitaniae* | 05 | | *Clavispora lusitaniae*** | | 05 | |
| *Candida kefyr* | 01 | | *Kluyveromyces marxianus*** | | 01 | |
| *Candida norvegensis* | 01 | | *Pichia norvegensis*** | | 01 | |
| *Candida pelliculosa* | 06 | | *Wickerhamomyces anomalus*** | | 06 | |
|  |  | |  | |  | |
| *Candida guilliermondii* | 22 | | *Meyerozyma guilliermondii*** | | 21 | |
|  |  | | ***Meyerozyma caribbica***** | | **01** | |
|  |  | |  | |  | |
| *Candida parapsilosis* (*sensu lato*)*** |  | | *Candida parapsilosis* (*sensu stricto*) | | 34 | |
|  | 69 | | *Candida orthopsilosis**** | | 31 | |
|  |  | | *Candida metapsilosis**** | | 03 | |
|  |  | | ***Lodderomyces elongisporus*** | | **01** | |
|  |  | |  | |  | |
| *Candida* spp. | **07** | | ***Clavispora lusitaniae***** | | **01** | |
|  |  | | ***Candida intermedia*** | | **03** | |
|  |  | | ***Candida haemulonii*** | | **02** | |
|  |  | | ***Candida duobushaemulonii*** | | **01** | |
| Total number of isolates | 300 | |  | | 300 | |

*Phenotypic identification of *C. albicans* and *C. dubliniensis* isolated from the oroesophageal cavity was performed by tolerance growth testing at 42°C and on hypertonic Sabouraud broth.

***Pichia kudriavzevii* = teleomorph of *Candida krusei*, *Clavispora lusitaniae* = teleomorph of *Candida lusitaniae*, *Kluyveromyces marxianus* = teleomorph of *Candida kefyr*; *Pichia norvegensis* = teleomorph of *Candida norvegensis*, *Wickerhamomyces anomalus* = teleomorph of *Candida pelliculosa*, *Meyerozyma guilliermondii* = teleomorph of *Candida guilliermondii*, *Meyerozyma caribbica* = teleomorph of *Candida fermentati*.

***The phenotypic method provided correct identification of *C. parapsilosis* complex without species discrimination.

Bold text indicates species for which the phenotypic and molecular methods produced discordant results.
